# Modeling of Non-Ferrous Metallurgy Waste Disposal with the Production of Iron Silicides and Zinc Distillation

**DOI:** 10.3390/ma15072542

**Published:** 2022-03-30

**Authors:** Alexandr Kolesnikov, Roman Fediuk, Mugahed Amran, Sergey Klyuev, Alexander Klyuev, Irina Volokitina, Aigul Naukenova, Shermakhan Shapalov, Akmaral Utelbayeva, Olga Kolesnikova, Aidana Bazarkhankyzy

**Affiliations:** 1 Department of “Life Safety and Environmental Protection”, M. Auezov South Kazakhstan University, Building B, Av. Tauke Khan, 5, Shymkent 160012, Kazakhstan; nas1970@mail.ru (A.N.); shermahan_1984@mail.ru (S.S.); akmutelbayeva@rambler.ru (A.U.); 2 Polytechnic Institute, Far Eastern Federal University, 690922 Vladivostok, Russia; fedyuk.rs@dvfu.ru; 3 Peter the Great St. Petersburg Polytechnic University, 195251 St. Petersburg, Russia; bazarkhankyzy.a@gmail.com; 4 Department of Civil Engineering, College of Engineering, Prince Sattam Bin Abdulaziz University, Alkharj 16273, Saudi Arabia; m.amran@psau.edu.sa; 5 Department of Civil Engineering, Faculty of Engineering and IT, Amran University, Amran 9677, Yemen; 6 Department of Theoretical Mechanics and Strength of Materials, V.G. Shukhov Belgorod State Technological University, 308012 Belgorod, Russia; klyuyev@yandex.ru (S.K.); klyuyevav@yandex.ru (A.K.); 7 Department of “Metallurgy and Mining”, Rudny Industrial Institute, Rudny 459120, Kazakhstan; irinka.vav@mail.ru; 8 Research Institute of New Chemical Technologies, L.N. Gumilyov Eurasian National University, Nur-Sultan 010008, Kazakhstan

**Keywords:** technogenic waste of non-ferrous metallurgy, clinker from welting, environmental pollution, thermodynamic modeling, degree of transition, zinc, iron, silicon, iron silicides

## Abstract

This paper presents studies on the possibility of utilization of technogenic waste from the metallurgical industry by the method of complex processing in order to reduce the anthropogenic load on the environment of the region with the example of the zinc silicate-magnetite-carbon system. The selected sample of clinker dump from welting was subjected to chemical and scanning electron microscopic analyses and thermodynamic modeling. Thermodynamic studies were carried out in the temperature range 1600–2200 K and pressure *p* = 0.1 MPa, modeling the process of electric melting of clinker from welting in an arc furnace using the software application Astra 4 developed at the Bauman Moscow State Technical University (Moscow, Russian Federation). As a result of the thermodynamic modeling, the optimal temperature range was established, which was 1800–1900 K. Thermodynamic studies established that it is possible to drive away zinc from the system under study by 99–100% in the entire temperature range under study. The maximum degree of silicon extraction (α_Si_) in the alloy is up to 69.44% at T = 1900 K, and the degree of iron extraction (α_Fe_) in the alloy is up to 99.996%. In particular, it was determined and proved that clinker waste from welting can act as a secondary technogenic raw material when it is processed as a mono mixture to produce iron silicides with a silicon content of 18 to 28%.

## 1. Introduction

Industrial waste located in dumps and containing in its chemical composition heavy non-ferrous metals and various compounds of silicon, calcium, aluminum and iron has a negative impact on the environment from the point of view of ecology, in particular on public health, flora and fauna [1,2,3,4,5,6,7,8]. However, based on its chemical composition, it is valuable and can serve as a secondary technogenic raw material for various kinds of industries, for example in the metallurgical, construction, chemical and other industries [9,10,11,12,13,14,15,16,17,18,19,20,21,22,23,24,25,26,27,28,29,30,31,32,33,34,35,36,37,38,39,40,41,42,43,44,45,46,47,48,49,50,51,52,53]. Thus, in Kazakhstan, in the process of extracting non-ferrous metals at a number of plants, since the 1920s and up to the present, in a number of areas, in particular, in the east Kazakhstan and Turkestan regions, there is a significant amount of clinker waste from the cultivation of various raw materials, which is now stored in dumps, occupying fertile lands and polluting the soil, surface and groundwater, the atmosphere, penetrating animal and human organisms through the migration of heavy metals along the food chain [9,10,11,12,13,14,15,16,17,18,19,20,21,22,23,24,25,26,27,28,29,30,31,32,33,34,35,36,37,38,39,40,41,42,43,44,45,46,47,48,49,50,51,52,53].

According to various estimates, at present, in the village of Achisai (Turkestan region), in the dumps, there are 4.5–5.7 million tons of zinc industry waste formed over the years of the Achisai polymetallic combine, which contains, according to various data, at least 102 thousand tons of zinc (Zn), 16 thousand tons of lead (Pb), 410 thousand tons of silicon (Si), 1.1 million tons of iron (Fe) and about 780–815 thousand tons of carbon (C). Despite this, clinker from welting is now considered only as raw material to extract carbon and obtain an iron-containing magnetic concentrate with the extraction of up to 80% of iron into it. At the same time, silicon, calcium, aluminum and non-ferrous metals are not extracted and pass into the flotation tails of the non-magnetic fraction, which are recommended for use in the production of building materials [8,39,40,41,42,43,44,45,46,47,48,49,50,51,52,53].

In different years, there have been many attempts to use clinker from welting. In particular, a study [54] showed the possibility of obtaining building materials and mineral wool from Achisai clinker, as well as the possibility of using it in road construction [55]. There is a well-known work on the processing of clinker welting slags from mine lead smelting by magnetic separation to obtain (25–30%) ferromagnetic concentrate with a content of 75–89% Fe and 1–1.5% Cu [56]. At the same time, the magnetic concentrate was used in the fusing of slag, in the charge of agglomeration of lead production, in the enrichment of oxidized copper ores (instead of cast iron shavings), and a coal concentrate with a content of 58%C was obtained from the non-magnetic fraction, which is recommended for use in welding by blowing into the furnace or granulating with recycled dust [57].

At the Elektrozink plant, an experiment was carried out on blowing clinker with compressed air (blowing off carbon) and feeding a coal–air mixture into the head of the Welz furnace. However, despite an increase in productivity (by 10%) and a decrease in coke consumption, the experiment was discontinued due to deterioration in the quality of Welz oxide due to contamination with ash and carbon [58].

The experience of processing rich clinker in Bulgaria is interesting [59]. Clinker containing (%): Cu—2.23; Zn—1.31; Pb—1.25; C—19.1; SiO_2_—20.0; S—4.47, as well as 200 g/t Ag and 12 g/t Au, is subjected to screening. Class +16 mm is shipped to copper smelters, the rest (−16 mm) is divided into heavy suspension, after which the heavy fraction is sent to the copper smelter, and the light fraction is used in Welz furnaces. At the same time, the extraction of copper into products for metallurgical processing is up to 93%.

For the processing of clinkers poor in precious metals, more complex technological schemes are used, with a combination of flotation and magnetic separation. Thus, foreign researchers were able to achieve the extraction of copper and gold into processed products up to 91.7% and silver—89.1%. At foreign enterprises, this makes it possible to obtain concentrates with a content of 1.5% Cu, 515–620 g/t Ag, with a content of Cu in the non-magnetic fraction of 0.05%, C—80% (Peru, La Oroya plant) or Cu—1.6%, Au—3.2 g/t and 544 g/t Ag (Japan, Aizu plant) [60,61].

For the extraction of non-ferrous metals from the clinkers of the UCCC and CHECZ in GINTSVETMET, a chloride-distilling method in a fluidized bed furnace has been developed [62]. The method was tested on a semi-industrial installation with an hourly capacity of 165 kg, for raw materials. The disadvantages of the method are the long duration of the process—5.5 h—a large consumption of concentrated CaCl_2_ solution—30% of the ore mass—as well as a relatively high residual content of Zn (0.5%) and Cu (0.25%) in the cinder.

The Kazakh Chemical Technological Institute (KazSSR) has developed a chloride method for processing UKCC clinkers in a tubular rotary kiln with a combination of chloride distillation of non-ferrous metals in the furnace [63]. Despite the fact that the economic effect of the developed method is USD ≈ 10 for each ton of clinker, the method cannot be considered rational, since it provides for the processing of the charge, in which the share of non-metallic components accounts for 55.9%.

In the 1990s, Yuzhpolymetal CJSC began work on the processing of Achisai clinker to obtain magnetic concentrate and coke, which never received a mass character, limiting itself to research experiments. However, the technological indicators of this process (including the extraction of non-ferrous metals) are not described in the special literature. None of the above methods have been implemented, and the clinker from the welting is not being disposed of at the moment and continues to pollute the environment of the region.

In the conducted experiments, using thermodynamic modeling based on the Astra 4 program, the possibility of complex utilization of clinker dump to reduce anthropogenic impact on the biosphere of the region was investigated using the example of the ZnO∙SiO_2_-FeO∙Fe_2_O_3_-C system with the production of iron silicides and zinc sublimates from it.

## 2. Materials and Methods

As the material under study, a sample taken from the clinker dump of zinc oxide ores was used. The studied technogenic waste in the form of clinker from welting was subjected to chemical [64], scanning electron microscopic [65] analyses and thermodynamic modeling of its processing by electric melting in an arc furnace to produce iron silicides and zinc-containing sublimates.

At the present stage, when solving many scientific and technical problems, the issues of studying high-temperature processes with physicochemical transformations, for example, combustion processes, play a significant role. The experimental methods of studying processes of this kind are usually expensive and often not feasible at all. Under these conditions, a computational experiment performed using a computer acquires special importance, which allows analyzing states and processes and drawing conclusions about the behavior of the objects under study based on model representations [66,67].

The main assumption, in this case, is the assumption of the existence of a local equilibrium in the system, which makes it possible to carry out calculations using the mathematical apparatus of equilibrium thermodynamics [66,67].

The main task of modeling thermodynamic equilibrium is to determine the phase and chemical composition, as well as the values of the thermodynamic parameters of the system under study [66,67].

Thermodynamic modeling of chemical and phase transformations in the system under study was carried out using the computer program Astra 4, which was developed by a group of scientists of the Bauman Moscow State Technical University and operates using the principle of maximum entropy [66,67].

The Astra software package is based on the principle of maximum entropy—a factor associated with the degree of ordering of the energy state of the microparticles that make up the working fluid. The laws of statistical physics allow us to find the number of discrete states that a specific (given) microstate implements. A comparison of this value with entropy allows us to establish that the latter is a measure of the probability of the state of the system. Therefore, maximum entropy corresponds to the equilibrium conditions of the considered set of particles of the working medium, i.e., the relationship between the probability of the state of the system and its entropy (S) allows us to formulate an extreme condition defining the state of the system by expressions:

S = S_max_ M_i_ = const, U_n_—const, v = const,

where: 

U_n_ is the total internal energy;

M_j_ is the mass of the working fluid;

v is the specific volume of the entire system.

The formulation of the thermodynamic modeling problem requires assigning two conditions for the equilibrium of the system under study with the environment. These conditions can be either numerical values of the thermodynamic characteristics of the equilibrium or functional relations between the parameters of this state. To describe the system itself as a material object, it is necessary to know only the content of the chemical elements forming it. Internal and interphase interactions are described by model thermodynamic relations, for the closure of which the properties of only individual substances—the equilibrium component—are used [66,67].

Due to its accessible formulation of the problem of various kinds of modeling, the developed Astra 4 complex promotes the use of the thermodynamic method for the study of various possibilities of the course of a wide variety of processes under conditions of various physicochemical states [66,67].

## 3. Results

In the course of the research, a chemical analysis of technogenic waste—clinker from welting—was carried out, the results of the study of which are given in Table 1.

Having studied the results of the chemical analysis, they approximately coincide with previous studies of clinker’s chemical composition with predominance in iron composition [9,39,68].

**Figure 1:**
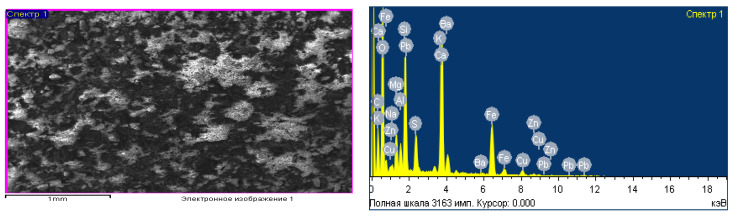
Micrography (**a**) and elemental analysis (**b**) of metallurgical waste.

Additionally, a sample of clinker from welting was analyzed on a scanning electron microscope of the brand JSM-6490l (Joel, manufactured in Japan) to obtain its elemental composition. The results of the studies are shown in Figure 1. From these results, it follows that the present waste in the form of clinker from welting in its composition contains elements such as zinc, lead, calcium, silicon, oxygen, iron, aluminum (which is also confirmed by the results of the chemical analysis carried out earlier) [9,39,68].

From various studies of scientists [9,39,58], it is known that clinkers from the welting of various kinds of materials contain Fe (iron) in the reduced state in the form of elemental (Fe) and oxide state (FeO, FeO∙Fe_2_O_3_), Zn (zinc) silicate form (ZnO∙SiO_2_), and carbon is present in the form of coke that did not have time to react in the welting of zinc oxide ores.

**Figure 2:**
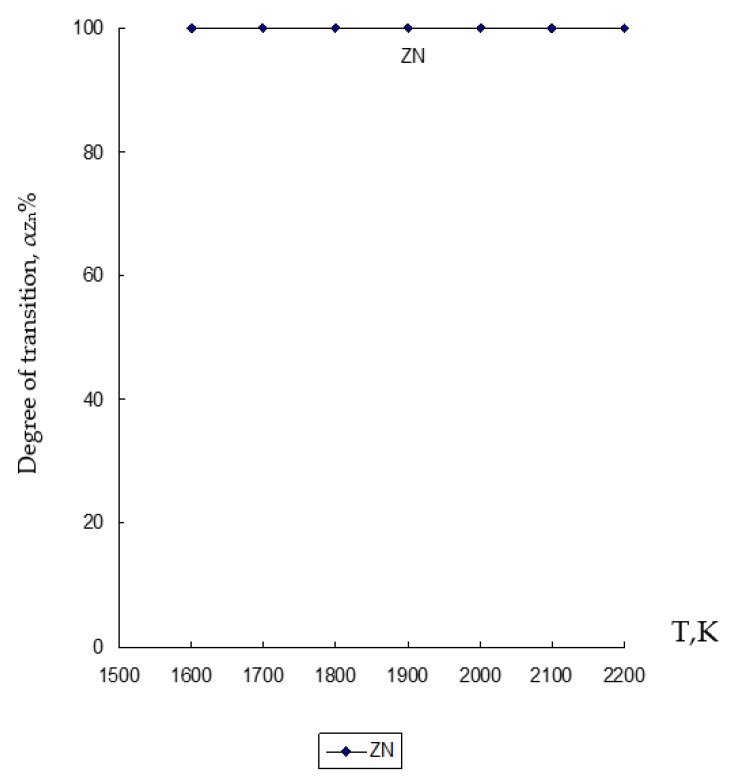
Influence of temperature (T) on the degree of distribution (α) of Zn in the ZnO∙SiO_2_-FeO∙Fe_2_O_3_-C system.

**Figure 3:**
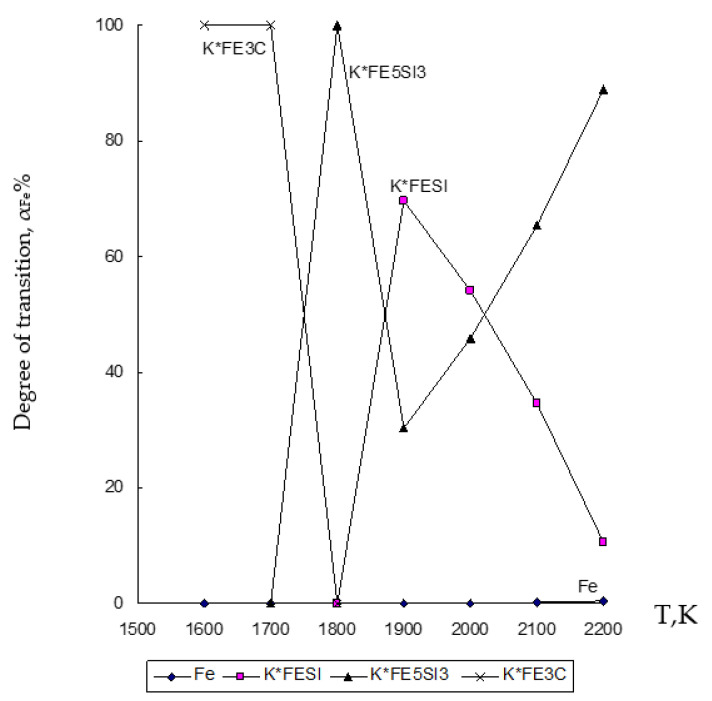
Influence of temperature (T) on the degree of distribution (α) of Fe in the ZnO∙SiO_2_-FeO∙Fe_2_O_3_-C system.

**Figure 4:**
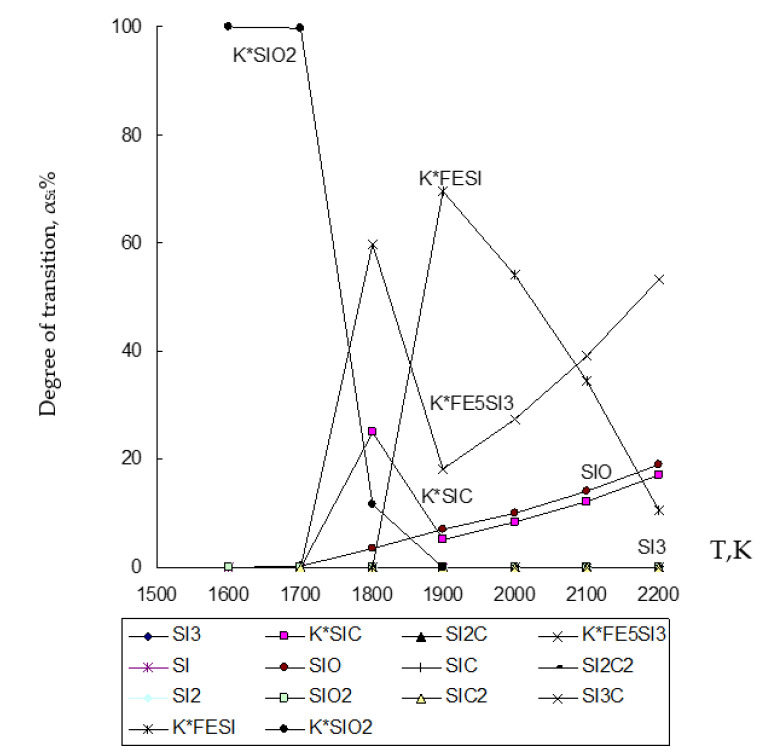
Influence of temperature (T) on the degree of distribution (α) of Si in the ZnO∙SiO_2_-FeO∙Fe_2_O_3_-C system.

Based on the results of determining the chemical composition of the waste under study given in Table 1 and previously obtained data by various scientists [9,68,69,70], chemical and phase interaction was simulated in the temperature range 1600–2200 K with a pressure of *p* = 0.1 MPa in the heterogeneous system under study ZnO∙SiO_2_-FeO∙Fe_2_O_3_-C, where the interaction of magnetite (FeO∙Fe_2_O_3_) with zinc silicate (ZnO∙SiO_2_) and carbon (C), which are contained in the clinker from Waelz process (Figure 2, Figure 3 and Figure 4, and Table 2).

In studies on the interaction in the ZnO∙SiO_2_-FeO∙Fe_2_O_3_-C system, the following chemical equation was adopted as the basic one:

3ZnO∙SiO2 + FeO∙Fe2O3 + 13C = 3 Zn + 3FeSi + 13CO.(1)

The results of modeling the chemical and phase interaction in the system ZnO∙SiO_2_-FeO∙Fe_2_O_3_-C are shown in Figure 2, Figure 3 and Figure 4 and in Table 2.

The effect of temperature on the distribution of zinc (Zn), iron (Fe), silicon (Si), carbon (C), oxygen (O_2_) in the ZnO∙SiO_2_-FeO∙Fe_2_O_3_-C system is characterized by the formation of seven elements and compounds: Zn, Fe, C, k*C (where k*- means condensed phase), Si, Si_2_, Si_3_, FeSi, Fe_5_Si_3_, k*Fe_3_C, k*SiC, CO and CO_2_.

## 4. Discussion

From the results obtained in the chemical and phase modeling carried out under the conditions of the ZnO∙SiO_2_-FeO∙Fe_2_O_3_-C system, it is clear that zinc (Zn) completely passes into the gas phase with a 100% degree of transition in the entire set temperature regime (Figure 2).

Figure 3 shows that in the ZnO∙SiO_2_-FeO∙Fe_2_O_3_-C system, iron (Fe) passes into an alloy with a transition degree of up to 99.996% for a compound formed as Fe_5_Si_3_ at 1800 K, and up to 69.58% for a compound formed as FeSi at 1900 K.

The degree of distribution of silicon (Si) in the ZnO∙SiO_2_-FeO∙Fe_2_O_3_-C system into iron-containing compounds of the condensed phase is shown in Figure 4. Figure 4 shows that the degree of Si transition to the alloy reaches up to 59.88% for the compound formed as Fe_5_Si_3_ at 1800 K, and up to 69.44% for the compound formed as FeSi at 1900 K.

Table 2 shows the results of the degree of distribution of (α) oxygen (O_2_) and carbon (C) in the ZnO∙SiO_2_-FeO∙Fe_2_O_3_-C system from temperature. Table 2 shows that oxygen is mainly distributed into compounds such as CO, CO_2_, k*SiO_2_. So, at T = 1900 K, oxygen is distributed into the following compounds: CO_2_ by 98.297%; SiO by 1.693%; CO by 0.01% and SiO_2_ by 0.00006%.

The degree of distribution of (α) carbon (C) in the ZnO∙SiO_2_-FeO∙Fe_2_O_3_-C system from temperature is mainly represented by such compounds as CO_2_ (from 54.025% to 98.721%), k*C (from 38.192% to 38.249%) and k*Fe_3_C by 7.714% (Table 2).

Based on the obtained results of the thermodynamic modeling, which are presented in Figure 2, Figure 3 and Figure 4, it follows that under the given temperature conditions in the conditions of the ZnO∙SiO_2_-FeO∙Fe_2_O_3_-C system, it is possible to almost completely extract Zn with its transfer to the gas phase with a transition degree (α_Zn_) equal to 100%. Simultaneously with this simulation, the conversion of iron (10.6–99.996%) and silicon (10.7–69.44%) into an alloy with the possibility of forming iron silicide compounds with a predicted Si content within 18–28% (which, according to GOST 1415-93 (ISO 5445-80), can be identified as ferrosilicon grades FS18, FS20 and FS25) under optimum conditions (1900–2000 K) of the studied temperature regime. The obtained results of the study of the disposal of clinker waste in order to reduce the anthropogenic impact on the biosphere of the region with the possibility of obtaining ferroalloy and zinc sublimates are new and can complement the ongoing research in this direction [8,24,25,26,27,28,39,71,72,73,74,75,76].

## 5. Conclusions

Based on the obtained results of studies of the possibility of complex utilization by processing technogenic waste in the form of clinker in order to reduce the anthropogenic impact on the biosphere of the region, the following conclusions can be drawn using the example of the ZnO∙SiO_2_-FeO∙Fe_2_O_3_-C system with the production of ferroalloy and zinc sublimates:

- from the clinker dump, it is possible to obtain a low-grade silicon-containing ferroalloy with a Si content in the range of 18–28% and Fe in the range of 73–82% and extract Zn into the gas phase in the range of 99–100% in the form of zinc sublimates in the optimal temperature range of 1800–1900 K;
- zinc contained in the clinker can be driven into the gas phase by 100% with further capture as zinc sublimates;
- technogenic waste—the clinker dump from rolling zinc oxide ores, according to its chemical and elemental compositions, can act as a secondary technogenic raw material for the metallurgical and chemical industries;
- modeling of clinker utilization by electric melting in an arc furnace will contribute to its processing and, accordingly, reduce the anthropogenic impact of its dump on the biosphere of the region with a multiplicative socio-ecological and economic effect.

## Figures and Tables

**Table 1:** Chemical composition of clinker blade from welting.

№	Compounds, Elements	Percentage Content, %
1	CaO	14.87
2	SiO_2_	18.12
3	MgO	2.81
4	Al_2_O_3_	4.75
5	Fe_2_O_3_	26.98
6	Zn	0.94
7	Pb	0.12
8	Cu	0.11
9	S	1.4
10	C	18.6
11	BaO	2.4
12	Other	8.9

**Table 2:** Influence of temperature (T) on the degree of distribution of (α) oxygen (O_2_) and carbon (C) in the ZnO∙SiO_2_-FeO∙Fe_2_O_3_-C system.

T, K	1600	1700	1800	1900	2000	2100	2200
Compounds, %	Degree of distribution (α) of oxygen (O_2_), %						
CO	0.0219	0.0102	0.0137	0.0102	0.0063	0.0041	0.0028
CO_2_	53.802	53.864	93.813	98.297	97.628	96.743	95.624
C_2_O	0	0	0	0	0.0000003	0.0000008	0.0000021
SiO	0.003	0.054	0.815	1.693	2.366	3.253	4.373
SiO_2_	0	0.0000003	0.0000038	0.0000675	0.0000073	0.0000080	0.0000880
k*SiO_2_	46.174	46.072	5.358	0	0	0	0
Amount, %	100	100	100	100	100	100	100
	Degree of distribution (α) of carbon (C), %						
k*C	38.249	38.192	0	0	0	0	0
C	0	0	0	0	0	0	1.08136^−7^
CO	0.011	0.005	0.006	0.005	0.003	0.002	0.001
CO_2_	54.025	54.088	94.202	98.721	98.037	97.144	96.021
k*SiC	0	0	5.791	1.273	1.959	2.852	3.975
SiC	0	0	0	0	0	0	3.4492^−7^
Si_3_C	0	0	0	0	0	3.50517^−7^	1.68534^−6^
Si_2_C	0	0	1.66238^−7^	2.26741^−6^	1.92375^−5^	0.00013	0.0007
SiC_2_	0	0	4.66156^−7^	4.67117^−6^	3.70176^−5^	0.00024	0.0013
Si_2_C_2_	0	0	0	0	0	2.9913^−7^	2.55166^−5^
C_2_O	0	0	0	0	2.56567^−7^	7.6273^−7^	0.0000025
k*Fe_3_C	7.714	7.714	0	0	0	0	0
Amount, %	100	100	100	100	100	100	100

## Data Availability

The data presented in this study are available on request from the corresponding author.
